# On the Development of Harmony, Turbulence, and Independence in Parent–Adolescent Relationships: A Five-Wave Longitudinal Study

**DOI:** 10.1007/s10964-016-0627-7

**Published:** 2017-01-02

**Authors:** Hana Hadiwijaya, Theo A. Klimstra, Jeroen K. Vermunt, Susan J. T. Branje, Wim H. J. Meeus

**Affiliations:** 1 Department of Developmental Psychology, Tilburg University, Postbox 90153, Tilburg, LE 5000 The Netherlands; 2 Department of Methodology and Statistics, Tilburg University, Postbox 90153, Tilburg, LE 5000 The Netherlands; 3 Research Centre Adolescent Development, Utrecht University, Postbox 80140, Utrecht, TC 3508 The Netherlands

**Keywords:** Parent–adolescent relationship, Adolescent development, Individual differences, Person-centered approach

## Abstract

The separation-individuation, evolutionary, maturational, and expectancy violation-realignment perspectives propose that the relationship between parents and adolescents deteriorate as adolescents become independent. This study examines the extent to which the development of adolescents’ perceived relationship with their parents is consistent with the four perspectives. A latent transition analysis was performed in a two-cohort five-wave longitudinal study design covering ages 12–16 (*n* = 919, 49.2% female) and 16–20 (*n* = 392, 56.6% female). Generally, from 12 to 16 year adolescents moved away from parental authority and perceived increasing conflicts with their parents, whereas from 16 to 20 years adolescents perceived independence and improved their relationships with parents. Hereby, we also identified substantial patterns of individual differences. Together, these general and individual patterns provide fine-grained insights in relationship quality development.

## Introduction

Distress in family relationships often increases as adolescents strive for more autonomy and independence (Laursen and Collins 2009). So far, research has mainly focused on general patterns of relationship quality development, while individual differences in development received less attention. However, whereas some adolescents might perceive distress in their relationship development, others might not (Arnett 1999). It could also be that those who perceive distress succeed in restoring the relationship quality with their parents by the end of adolescence, whereas others fail (e.g., Laursen et al. 2010). This study provides a comprehensive perspective on changes in parent–adolescent relationship quality by examining both general and individual developmental patterns. For this purpose, a person-centered (i.e., latent transition) approach was applied to a two-cohort five-wave longitudinal study design covering ages 12–16 and 16–20.

### Theoretical Perspectives on Parent–Adolescent Relationship Development

Various theoretical perspectives address change in parent-adolescents relationship quality across adolescence. Within the literature on parent–adolescent relationship development, the *separation-individuation,* the *evolutionary,* the *maturational,* and the *expectancy violation-realignment* perspectives particularly stand out (see review Branje et al. 2012). The separation-individuation perspective poses that hormonal changes in puberty are the main force driving adolescents to separate themselves from their parents to become autonomous and independent individuals (Blos 1967). The evolutionary perspective also emphasizes the role of puberty, and suggests that the distance between adolescents and parents increases as adolescents strive for individuation to find a sexual partner (Steinberg 1989). The related maturational perspective suggests that adolescents strive to change the unilateral hierarchical relationship with their parents to a more cooperative and egalitarian one as a result of their cognitive development during adolescence (Laursen and Collins 2009; Youniss and Smollar 1985). Parents, however, may resist these changes, resulting in more distress in their relationships (i.e., less closeness, more conflicts). Finally, the expectancy violation-realignment perspective relates to previous perspectives by proposing that discrepancies in autonomy expectations lead to disturbances in parent–adolescent relationships, but that these relationships eventually realign or improve by the end of adolescence (Collins and Luebker 1994).

All four perspectives emphasize the role of independence, equality, and distress in relationship quality development. However, they seem to disagree on how increasing relationship distress would affect relationship quality. Specifically, both separation-individuation and evolutionary perspectives seem to propose that increasing distress in the separation process would eventually cause a wedge between parents and adolescents, but both are silent about potential restoration of relationships in the second half of adolescence. The maturational and realignment perspectives do seem to suggest that satisfactory relationships can be (re)established by the end of adolescence, as distress is thought to diminish once the relationship is restructured. Thus, despite the evident similarities between the perspectives, there are some discrepancies in terms of the state of the parent–adolescent relationship by the end of adolescence.

### Empirical Evidence Concerning Relationship Development

Features of independence, equality, and distress overarch many conceptualizations of parent-adolescent relationship quality (e.g., De Goede et al. 2009; Steinberg and Silk 2002), and are reflected in Furman and Buhrmester’s (1985) three-component operationalization of close relationships. These components are *support, negative interaction,* and *power*. Specifically, support refers to nurturance and prosocial behavior, negative interaction includes disagreements and antagonism, and power represents authority versus equality. When examining relationship development as described by the previously discussed theoretical perspectives, the power component relates to processes of independence and equality, whereas both low levels of support and high levels of negative interaction relate to distress.

Several longitudinal studies have examined developmental trends in parent-adolescent relationship quality using the aforementioned key components. For example, De Goede et al. (2009) examined all three key components and showed that across adolescence parental authority diminished, parental support temporarily decreased, and negative interaction temporarily increased. Likewise, other studies have found that parental authority decreased over time, indicating that adolescents perceived more independence from their parents (e.g., Darling et al. 2008; Loeber et al. 2000). Relatedly, distress in parent–adolescent relationships increased from early to middle adolescence, and decreased thereafter (e.g., Keijsers et al. 2011; Tsai et al. 2013; van Wel 1994). In short, adolescents’ increase in their desire for independence and equality toward parents seems to be coupled with a temporary increase in distress (i.e., a reverse U-shape pattern). This implies that relationship quality can be restored by the end of adolescence.

### Individual Differences and Constellations of the Key Relational Elements

Although prior studies demonstrated temporary distress in parent–adolescent relationships as adolescents become independent, there is a lack of detailed knowledge on individual differences in these developments using all key relational components. Specifically, most longitudinal studies applied *variable-centered* approaches that focused primarily on single components of relationship development and/or examined general changes that are valid for the entire sample, but neglected heterogeneity in development. Such studies thus largely ignore individual differences in development. This is a limitation because obviously not all individuals will perceive increasing distress in early adolescence or positively realign the relationship quality with their parents by the end of adolescence. In fact, many studies already have demonstrated that only a subgroup of adolescents perceive increasing distress in their relationship with their parents across adolescence (e.g., Choe et al. 2014; Seiffge-Krenke et al. 2010; Skinner and McHale 2016; Timmons and Margolin 2015). These studies, however, do not use all of the key components support, negative interaction, and power. Specifically, constellations of relationship components rather than using singular components only would provide a better understanding of the exact quality of a relationship. This is because the interpretation of relationship quality depends on the relational aspects included. For example, the interpretation of a relationship quality with high levels of power would depend on the levels of both support and negative interaction. That is, high power could represent a cooperative authoritarian relationship when combined with high levels of support and low levels of negative interaction; whereas high power may illustrate a destructive hierarchical relationship while combined with low levels of support and high levels of negative interaction. This shows the importance of considering several relationship quality dimensions simultaneously. Thus, we argue that parent–adolescent relationship development should ideally be examined by investigating how development varies across individuals using all key relational components.

A *person-centered approach* can address individual differences in relationship quality and its development using all key relational elements*.* First, this approach generates constellations of parent–adolescent relationship components *within individuals*. An example of one of these constellations is a harmonious relationship profile in which adolescents perceive high levels of parental support, low levels of conflicts with their parents, and low levels of parental power. Second, this approach allows the examination of *within-individual changes* of relational constellations across consecutive measurement occasions. Consider, for example, that adolescents in a harmonious relationship profile may remain or change into another profile across years (i.e., within-individual changes of component constellations). Using this approach could thus provide detailed insights in both individual differences in relationship quality and individual differences in the development with each relationship quality. We aim to address these two matters using a previously identified relationship typology and analytical procedure that we will describe below.

First, previous research demonstrated within-individual differences in parent–adolescent relationship quality by identifying a relationship typology based on constellations of the key relationship components of Furman and Buhrmester (1985). This research revealed four profiles representing *harmonious* (48% of the sample; high on support, low on negative interaction, and moderate on power), *average* (38%; moderate on support, negative interaction, and power), *turbulent* (9%; low on support, high on negative interaction and power), and *detached* (5%; low on all components) relationship quality (Hadiwijaya et al. 2015). They were replicable and showed distinctive patterns of associations with psychopathology and personality. Importantly, the harmonious, average, and turbulent profiles seemed to represent a substantial proportion of the sample (i.e., more than 5%). Therefore, these three profiles can provide a starting point to examine individual differences in relationship quality development.

Note, however, that we do not regard the aforementioned three profiles as perfect distinct categories, but the use of profiles can be seen as *one way* to explore patterns of individual differences or heterogeneity in relationships. Specifically, profiles are fuzzy because the borders between relationship profiles are not clearly separated (e.g., Asendorpf et al. 2001). In other words, there is an area of classification inaccuracy at the borders between the profiles. Recent procedures, however, are able to adjust for potential inaccuracies and thereby account for such fuzziness (e.g., Vermunt 2010). Using profiles adjusted for inaccuracy would be a valid approach to examine patterns of individual differences in relationships. However, because the sample specifity of this procedure, we could also identify profiles different from aforementioned obtained profiles when using a different sample.

Second, the use of latent transition analysis (LTA; Vermunt et al. 2008) can reveal within-individual differences in adolescents’ perceived relationship quality development. This method generates relationship profiles using a set of components, identifies the number of adolescents in various profiles at every measurement occasion, and estimates the extent to which adolescents remain in their profile or change into another (e.g., Vermunt et al. 2008). For instance, it can identify the extent to which adolescents from a harmonious relationship change into an average relationship and the extent to which they change into a turbulent relationship. Thereby, individual differences can be uncovered in the extent to which distress in the parent–adolescent relationship is perceived. Relatedly, it can reveal the extent to which older adolescents change from a turbulent relationship into an average-quality or harmonious relationship, thereby demonstrating individual differences in relationship restorations (i.e., improvements). LTA is therefore ideal for identifying the extent to which adolescents change from a certain relationship (i.e., profile) into another over time, and for examining which particular adolescents perceive distress in the relationship with their parents and achieve satisfactory relationship realignment by the end of adolescence.

The LTA is a crucial procedure to identify individual differences in relationship quality and the developments of these individuals within each relationship quality by using constellations of all relational key components (i.e., support, negative interaction, and power). Specifically, previous person-centered longitudinal studies (e.g., Choe et al. 2014; Seiffge-Krenke et al. 2010) particularly examined the extent to which adolescents differ in the baseline levels and in the developmental trajectories of a certain relationship aspect across years (i.e., examining support, negative interaction, and/or power separately). Despite the importance of the findings, a singular classification into a relational trajectory provides fewer nuances in developmental differences than a procedure that generates the likelihood of individuals changing into a certain relationship quality for each consequent year. In addition, previous studies lack information about parent–adolescent relationships’ quality using all three key components. The use of LTA can overcome both issues by constellating relationship profiles using all three components and examine the extent to which adolescents change from a certain relationship quality profile into another profile from year to year.

The present study will use such a person-centered approach to examine the extent to which parent–adolescent relationship quality development is consistent with the separation-individuation, evolutionary, maturational, and realignment perspectives. We will pursue this goal by using a LTA. First, we aim to examine typical relationship developments by exploring change and stability in the prevalence of relationship quality profiles across the years. Second, we aim to identify the atypical developments by investigating individual patterns that explain the changes in prevalence of profiles (i.e., patterns of adolescents changing from one profile to another).

## Study Hypotheses

The four prominent theoretical perspectives predict an (temporary) increase of distress in relationships once individuals enter adolescence. Therefore, we expect an increase in the prevalence of the turbulent relationship profile and a decrease in the prevalence of harmonious and average relationship profiles in early-to-middle adolescence (i.e., ages 12–16). Relatedly, we anticipate that early-to-middle adolescents will be relatively more likely to remain in, or change to, a relationship in which they perceive increasing distress and hierarchy (i.e., a turbulent relationship profile).

Furthermore, the maturational and realignment perspectives seem to be relatively similar in proposing an egalitarian and satisfactory relationship establishment by late adolescence, whereas the separation-individuation and evolutionary perspectives are silent about potential relationship restorations. Hence, we expect an increase in the prevalence of both harmonious and average relationships and a decrease in the prevalence of turbulent relationships from middle-to-late adolescence (i.e., age 16–20). Thus, we anticipate that middle-to-late adolescents will generally be relatively more likely to remain in, or change to, a relationship with less distress and more equality (i.e., harmonious or average relationship profiles).

Next to these general or typical patterns, we also tentatively expect a considerable proportion of adolescents to exhibit developmental patterns differing from aforementioned typical developmental patterns. We will examine the individual differences and potential atypical patterns in an exploratory manner since no other developmental study has addressed this issue.

## Method

### Procedure

Data for the current study were collected as part of a longitudinal research project titled Conflict and Management of RElationships in The Netherlands (CONAMORE). The local institutional review board granted approval for this project. Participants were recruited from various high schools in the province of Utrecht, The Netherlands. Both adolescents and their parents received an invitation letter describing the research project and goals. The letter also provided information on how to decline from participation. More than 99% of the approached adolescents signed the informed consent form and thus agreed to participate in the study. Confidentiality of responses was guaranteed to all participants. Adolescents completed the questionnaires at school or at home at the annual measurement waves during which verbal and written instructions were offered. Instructions pertained to reading of the questionnaires, filling out of the answer categories, and time available to complete the various questions. For every wave they participated in, adolescents received a reward of €10 (approximately US$ 11).

### Participants

In the present study, we used the first five measurement waves with a one-year interval between each of these waves. Specifically, the additional sixth wave took place four years after the fifth wave. Consequently, including this wave would provide less accurate transitions patterns across years. Therefore, we decided to include these first five consecutive measurement waves only. The study sample (*N* = 1311) was divided into two age groups: an early-to-middle adolescent cohort (*n* = 919; 49.3% female, M_age_ = 12.4 years, SD = 0.57 at the first measurement wave) and a middle-to-late adolescent cohort (*n* = 392; 56.7% female, M_age_ = 16.7 years, SD = 0.81 at the first measurement wave). Thus, we use a two-cohort five-wave longitudinal study design covering ages 12–16 and 16–20.

The early-to-middle adolescent cohort included 467 boys (50.8%) and 452 girls (49.2%), whereas the middle-to-late adolescent cohort consisted of 170 boys (43.4%) and 222 girls (56.6%). At the first measurement wave, the vast majority of adolescents in both age groups reported that they lived with both parents (84.9%). Others reported living with their mother (7.7%) or elsewhere (e.g., with their father, with their biological parent and stepparent, or with other family members). Most participants identified themselves as Dutch (85.8%); others identified themselves as members of the most common ethnic minorities in The Netherlands (e.g., Surinamese, Antillean, Moroccan, Turkish). Overall, approximately 5.0% of the relationship quality data was missing across waves. Little’s (1988) missing completely at Random test indicated that these data were likely missing at random (χ^2^/df = 0.72; Bollen 1989). This suggests that adolescents with missing data were similar to those with complete data. For this reason, adolescents with missing data were included in the analyses using maximum likelihood estimation with incomplete data.

### Measurements

#### Relationship quality

Adolescents’ relationship quality with their mothers and fathers was measured separately using the Network of Relationships Inventory (NRI; Furman and Buhrmester 1985) (i.e., one NRI for each parent). Specifically, we measured adolescents’ perceptions of support received from their mothers and fathers, the intensity of negative interaction they perceived with their mothers and fathers, and the amount of power attributed to their mothers and fathers, separately. Participants were asked to indicate on a 5-point Likert scale (i.e., 1, “A little or not at all”, to 5, “More is not possible”) the degree to which each of the items described what they perceived. The support scale includes 12 items (e.g., “How much does your mother really care about you?”), the negative interaction scale includes six items (e.g., “Do you and your mother get on each other’s nerves?”), and the power scale includes another six items (e.g., “To what extent is your mother the boss in your relationship?”).

Internal consistency of all NRI scores was high. Specifically, alphas across waves were ≥.83 for scales referring to the mother–adolescent relationship and alphas ≥.87 for scales referring to the father–adolescent relationship. We collapsed the scores for adolescent–mother and adolescent–father relationship quality on each component, as our study aimed to identify general parent–adolescent relationship profiles. Principal component analysis showed that the underlying factors represented three relationship components rather than different adolescent–mother or adolescent–father relationship factors (results are available from the first author upon request). Also note that we identified measurement invariance of the NRI scales across age cohorts at the first and fifth measurement wave. This suggests that the NRI scales measure identical constructs in early-to-middle and middle-to-late adolescents. We present these results in Table 1.

**Table 1:** Measurement invariance tests for early and late adolescents’ perceived relationship quality with their fathers and mothers

Relationship quality	Wave	Model	χ^2^	df	χ^2^ */df*	CFI	TLI	RMSEA	BIC
NRI Mother	1	Baseline model	2181.87	540	4.04	0.88	0.87	0.07	68,947.99
		Metric invariance	2157.19	522	4.13	0.88	0.87	0.07	69,052.31
		Scalar invariance	2386.67	546	4.37	0.86	0.86	0.07	69,109.80
NRI Mother	5	Baseline model	2919.35	540	5.41	0.86	0.86	0.08	58,966.92
		Metric invariance	2798.96	522	5.36	0.87	0.86	0.08	58,974.75
		Scalar invariance	3055.35	546	5.60	0.86	0.86	0.09	59,060.17
NRI Father	1	Baseline model	2713.93	540	5.03	0.87	0.87	0.08	67,403.85
		Metric invariance	2699.76	522	5.17	0.87	0.86	0.08	67,518.09
		Scalar invariance	2923.57	546	5.35	0.86	0.86	0.08	67,570.68
NRI Father	5	Baseline model	3635.17	540	6.73	0.85	0.84	0.10	57,384.27
		Metric invariance	3500.12	522	6.71	0.85	0.84	0.10	57,376.72
		Scalar invariance	3722.56	546	6.82	0.84	0.84	0.10	57,429.16

*Note.* Comparisons of these three models demonstrated measurement invariance for early-to-middle and middle-to-late adolescents at the first and fifth measurement wave. Specifically, the baseline model is without any equality constrains and tests how the three relational constructs (i.e., support, negative interaction, and power) are operationalized for early-to-middle and middle-to-late adolescents. The metric invariance model only constrained the factor loadings to be equal across early-to-middle and middle-to-late adolescent cohort, whereas the intercepts are allowed to differ. This model tests whether early-to-middle and middle-to-late adolescents attribute the same meaning to the latent relationship constructs. The scalar invariance model constrained both the loadings and intercepts of the early-to-middle and middle-to-late adolescents to be equal. This model tests whether the meaning of the relationship constructs are equal in both cohorts. Although there were statistically significant chi-square differences between the models, the differences in CFI and RMSEA values are small (ΔCFI < .010 and ΔRMSEA < .015). Therefore, it is concluded that the NRI measures identical adolescent-mother and adolescent-father relationship constructs in early-to-middle and middle-to-late adolescents.

### Data Analyses

#### Main analyses

To answer our research questions, an LTA was performed in Latent GOLD version 5.0 (Vermunt and Magidson 2013). LTA is a longitudinal extension of latent profile analysis (LPA). LPA aims to identify unobserved classes or profiles of individuals in a population using a set of observed variables at one time point. Individuals are grouped into profiles, of which each contains individuals who are similar to each other and different from those in other profiles (Magidson and Vermunt 2004). To examine the extent to which individuals may change from one profile to another profile over time, LPA can be extended to LTA. LTA generates transition probabilities for profile membership over time (e.g., Vermunt et al. 2008). It can thus provide information on (i) the differences in relationships between individuals by producing relationship profiles using configurations of components (i.e., support, negative interaction, and power) and (ii) differences in the within-individual developments by generating transition probabilities between profiles over time.

The current LTA used five-wave data to identify relationship profiles and to provide information about changes in profile prevalence. Additionally, LTA generated estimates of initial classification probabilities and transition probabilities for adolescents moving from one profile to another (e.g., Vermunt et al. 2008). Initial classification probabilities reflect the probability of an adolescent belonging to a certain profile at baseline (i.e., the first wave of the current study). Transition probabilities refer to the probability of an adolescent moving to profile Y on the next measurement wave (e.g., Wave 2) conditional on having been in profile X on the previous wave (i.e., Wave 1).

Furthermore, transition probabilities between profiles may differ by measurement time (e.g., from the first to the second versus the third to the fourth and fifth measurement wave), gender (e.g., boys versus girls), and/or age cohort (i.e., early-to-middle adolescents versus middle-to-late adolescents). Therefore, measurement wave, gender, and cohort were included in the model as moderator variables. To examine potential differences in transition probabilities, we compared the fit of LTA-models with and without these moderator variables and the two-way interactions among these variables (i.e., time by gender, gender by age cohort, and age cohort by time). Specifically, if the LTA-model without any of the moderator variables has the best fit, then transition probabilities of adolescents who remain or change into a certain profile are similar for each measurement wave, gender, and age cohort. However, if the LTA-model with, for example, age cohort has the best fit, then transition probabilities differ for early-to-middle and middle-to-late adolescents.

We used two most commonly used criteria to select the best (and therefore final) latent transition model solution. First, the Bayesian information criterion (BIC; Schwarz 1978) should be the lowest, as this indicates an improvement in model fit. Second, the profile solution should be theoretically meaningful and parsimonious. That is, additional profiles should make theoretical sense and not be redundant with profiles that were already present in solutions that included fewer profiles (i.e., were more parsimonious). Several additional analyses were also included to clarify the main findings. We describe these analyses throughout the results section.

## Results

### Parent–Adolescent Relationship Profiles

#### Latent transition analysis: model selection

In total, we tested seven LTA models: One model without and six models with moderator variables. Models with moderator variables included the variables time (wave 1, 2, 3, 4, or 5), gender (male or female), age cohort (early-to-middle or middle-to-late adolescents), and two-way interactions among these variables (time by gender, gender by age cohort, and age cohort by time). We tested all models for up to six profiles. Only the profile solutions of the model moderated by cohort had lower BIC-values than the profile solutions of the other models for the 2-profile up to the 6-profile solutions. This suggested that the model moderated by cohort had the best fit-parsimony balance and that transition probabilities among profiles were *different* for early-to-middle versus middle-to-late adolescents. Therefore, we continued with the latent transition model moderated by cohort.

Next, we examined the profiles of the latent transition model moderated by cohort to determine the number of latent profiles. Solutions up to six latent profiles led to lower BIC-values, suggesting that each additional profile contributed to model fit improvement. However, when examining these profiles, the five-profile solution appeared to be the most parsimonious and theoretically meaningful. Specifically, the sixth-class of the six-profile solution of the early cohort sample was too small (<5%), thereby indicating a rare relationship profile for this subsample. The four-profile solution showed a worse model fit than the five-profile solution and missed two unique profiles that the five-profile solution did provide. Therefore, we selected the five-profile solution as the final one.

Figure 1a displays the profiles for the two-profile up to the five-profile solution. As can be seen this figure, the five-profile solution included two unique classes that the four-profile solution did not provide (i.e., class four and five in the five-class solution). However, this solution also included two classes that were already present in the four-profile solution and that were very similar to each other (i.e., class two and three in the five-class solution). Specifically, these two classes were similar to each other in terms of levels of relationship quality dimension and individual transitions. Keeping these classes separated thus seemed to provide little additional information, as they were relatively identical. For that reason, we decided to *merge* the fourth and fifth class of the five-profile solution for subsequent analyses in an effort to not lose the unique classes of this solution and to increase model simplicity (Hennig 2010). Figure 1b displays this integrated five-profile solution. The final model thus represented five profiles integrated into four profiles, with developmental transitions being different for early-to-middle and middle-to-late adolescents.

**Fig. 1 a:**
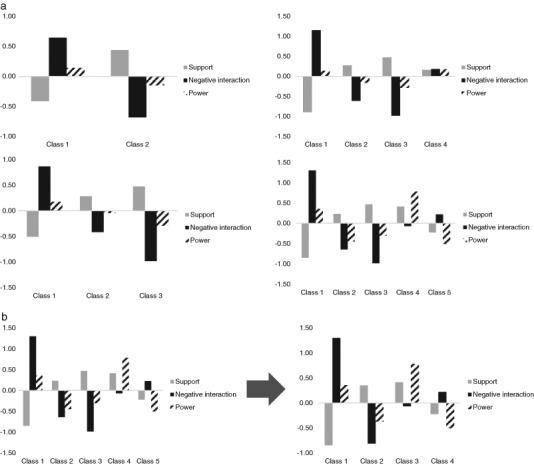
Parent–adolescent relationship profiles for latent transition solutions up to five classes based on adolescents’ perceived support, negative interaction, and power in the relationship with their parents (*N* = 1311). **b** Integrated four-class solution profiles of parent–adolescent relationships based on adolescents’ perceived support, negative interaction, and power in the relationship with their parents (*N* = 1311). The means of the integrated profile were calculated using the weighted means of the first and second profiles of the five-class solution

**Fig. 1b:**
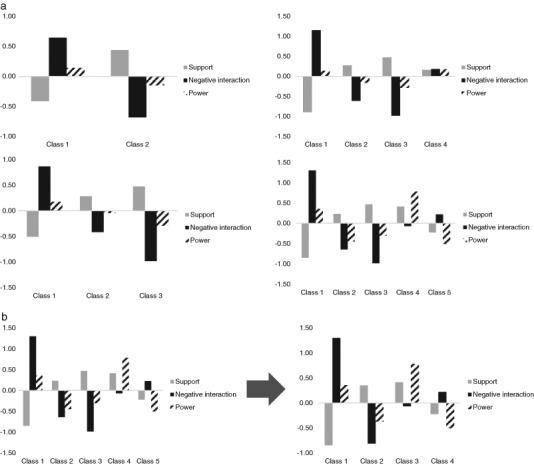
Integrated four-class solution profiles of parent–adolescent relationships based on adolescents’ perceived support, negative interaction, and power in the relationship with their parents (*N* = 1311). The means of the integrated profile were calculated using the weighted means of the first and second profiles of the five-class solution

Note that we also obtained similar relationship profiles when examining adolescents’ relationship quality with their mothers and fathers, separately. We examined these using the six key relational dimensions of adolescent-mother (i.e., adolescents’ reports on support, negative interaction, and power in the relationship with their mother) and adolescent-father (i.e., adolescents’ reports on support, negative interaction, and power in the relationship with their father). The LTA five-profile solution based on these six key dimensions revealed five *adolescent-mother* and *adolescent-father profiles* that were similar to our current five profiles in which we used the three key dimensions of *adolescents-parents* (i.e., the collapsed scores of adolescents’ reports on support, negative interaction, and power in the relationship with their mother and father). This suggests that merging mother-adolescent and father-adolescent relationship components into a generic parent-adolescent relationship quality leads to similar results as compared to studying mother-adolescent and father-adolescent relationship components separately in our sample using a five-class solution. Figure 1 of the supplemental material illustrates the profiles based on relationship quality dimensions of fathers and mothers, separately.

#### Latent transition analysis: relationship profiles

We labelled the four parent–adolescent relationship profiles as *turbulent, harmonious, authoritative, and uninvolved-discordant* (displayed in Fig. 1b from left to right, respectively). Adolescents in a harmonious relationship (37%) perceived high levels of support and low levels of power and negative interaction. Those who perceive an authoritative relationship (22%) reported high levels of support and power and moderate levels of negative interaction. Adolescents who perceive an uninvolved-discordant relationship (21%) reported low levels of parental support and power and high levels of negative interaction, whereas those who perceive a turbulent relationship (20%) reported particularly low levels of support and high levels of and negative interaction and power.

Next, we conducted an ANOVA to compare the differences in relationship quality between the profiles, while controlling for classification inaccuracy of the relationship profiles using a three-step procedure. For more information about this three-step procedure, please see Vermunt (2010). Table 2a illustrates the mean scores of individuals classified in the four relationship profiles on support, negative interaction, and power. This table shows that means on the three relationship quality dimensions were significantly different for all profiles. This table also displays the number of adolescents in each of the profiles. Moreover, we performed additional analysis on these profiles to examine whether parents and adolescents perceived the quality of their mutual relationship similarly. Hereby, we used the data of a subgroup of the parents (*N* = 308) from the early-to-middle adolescent cohort. Specifically, only these parents (and not those of the other 1003 participants) reported the extent to which they provided support and expressed power to their children. For this purpose, they filled out the Network of Relationships Inventory (Furman and Buhrmester 1985) at the second measurement wave only. Using these data, we examined whether parental levels of support and power significantly differed across each of the four relationship profiles as reported by adolescents. Table 2b show these results. It seemed that these profiles do not significantly differ on relationship quality as perceived by adolescents’ parents. For instance, parents from adolescents in a harmonious relationship perceived a similar relationship quality when compared to parents whose adolescent children perceived an authoritative, uninvolved-discordant, or turbulent relationship. This suggests that parents and adolescents perceived their relationships differently since they do not report distinct relationship quality patterns as their adolescent children do. Therefore, it should be kept in mind that our findings reflect *adolescent perceptions* of the relationship with their parents.

**Table 2a:** Three-step ANOVA total sample mean comparisons of relationship types at the first wave

Relationship quality	Harmonious	Authoritative	Uninvolved-discordant	Turbulent	Total	Wald value
	(*n* = 486)	(*n* = 285)	(*n* = 277)	(*n* = 263)	(*N* = 1311)	
	*M* (SD)	*M* (SD)	*M* (SD)	*M* (SD)	*M* (SD)	
Support	3.64 (0.61)^a^	3.70 (0.44)^b^	3.27 (0.50)^c^	2.87 (0.70)^d^	3.45 (0.64)	1815.08*
Negative interaction	1.08 (0.10)^a^	1.45 (0.26)^b^	1.58 (0.25)^c^	2.14 (0.66)^d^	1.48 (0.50)	534.68*
Power	2.41 (0.65)^a^	3.02 (0.50)^b^	2.14 (0.38)^c^	2.62 (0.75)^d^	2.56 (0.67)	350.62*

*Note.* *p < .001. Different superscripts represents significant mean-levels differences between relationship profiles. Profiles with different superscripts across rows differ from one another with regard to relationship quality. Post-hoc tests were Bonferroni corrected with *α* = 0.004, in which we divided the usual critical *p-*value of .05 in a two-tailed test by six (i.e., the total number of profile comparisons). Comparisons of classes on relationship quality were controlled for gender and age. For these comparisons, we used the total sample of adolescents (*N* = 1311).

**Table 2b:** Non-significant differences in perceived relationship quality by mothers and fathers at the second measurement wave

Relationship quality	Harmonious	Authoritative	Uninvolved-discordant	Turbulent	Total	Wald value
	(*n* = 140)	(*n* = 57)	(*n* = 49)	(*n* = 62)	(*N* = 308)	
	*M* (SD)	*M* (SD)	*M* (SD)	*M* (SD)	*M* (SD)	
Mother report on adolescent						
Support	3.36 (0.40)^a^	3.46 (0.42)^a^	3.31 (0.37)^a^	3.32 (0.43)^a^	3.36 (0.41)	6.03
Power	1.54 (0.33)^a^	1.57 (0.40)^a^	1.54 (0.39)^a^	1.62 (0.44)^a^	1.56 (0.38)	1.63
Father report on adolescent						
Support	3.23 (0.45)^a^	3.23 (0.40)^a^	3.15 (0.43)^ab^	3.08 (0.48)^bc^	3.19 (0.45)	6.79
Power	1.67 (0.39)^a^	1.71 (0.39)^a^	1.67 (0.35)^a^	1.72 (0.38)^a^	1.69 (0.38)	1.21

*Note.* Different superscripts represents significant mean-levels differences between samples. Samples with different superscripts across rows differ from one another with regard to relationship quality. Post-hoc tests were Bonferroni corrected with *α* = 0.004, in which we divided the usual critical *p-*value of .05 in a two-tailed test by six (i.e., the total number of profile comparisons). Comparisons of classes on relationship quality were controlled for gender and age. Please note that there was only limited data on paternal and maternal reports of the relationship quality with their children. That is, such data was only available for 23% of our total sample, on one measurement occasion, and on two out of three relational components.

### Stability and Change in Relationship Development

**Fig. 2:**
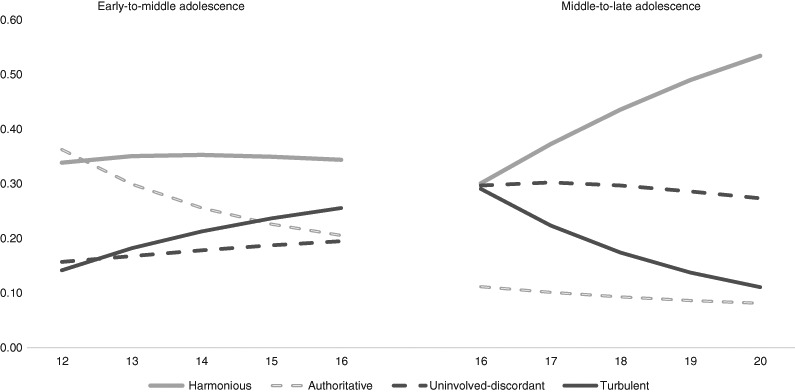
Parent–adolescent relationship percentage rates of early-to-middle (*n* = 919) and middle-to-late (*n* = 392) adolescents across five years

We performed an omnibus test using a logistic regression analysis to examine the overall changes across time in profile prevalence (with time as predictor and profile as outcome). Hereby, we also controlled for classification inaccuracy by using a three-step procedure (e.g., Vermunt 2010). The test revealed significant overall changes in profile prevalence during early-to-middle and middle-to-late adolescence separately (Wald-value = 119.76, *p* < .05 for the early cohort and Wald-value = 106.53, *p* < .05 for the late cohort). Figure 2 presents these prevalence patterns across waves. To follow up on the omnibus test, we also performed post-hoc tests by calculating the *z*-values and confidence levels of each profile in each wave to examine the differences in prevalence rates between waves and cohorts. Table 3 displays the prevalence of each profile in each wave and indicates whether the prevalence differed significantly between and within the cohorts.

**Table 3:** Size of parent–adolescent relationship profiles for early-to-middle and middle-to-late adolescents across five waves

Wave	Harmonious		Authoritative		Uninvolved-discordant		Turbulent	
	*n*	%	*n*	%	*n*	%	*n*	%
Early-to-middle adolescents (*n* = 919)								
1	311	0.34^a^	333	0.36*^a^	144	0.16*^a^	130	0.14*^a^
2	322	0.35^a^	275	0.30*^ab^	154	0.17*^a^	167	0.18^ab^
3	324	0.35*^a^	235	0.26*^b^	164	0.18*^a^	196	0.21^ab^
4	321	0.35*^a^	208	0.23*^b^	172	0.19*^a^	218	0.24*^b^
5	316	0.34*^a^	189	0.21*^b^	179	0.19^a^	235	0.26*^b^
Middle-to-late adolescents ( *n* = 392)								
1	118	0.30^a^	44	0.11*^a^	116	0.30*^a^	114	0.29*^a^
2	146	0.37^ab^	40	0.10*^a^	119	0.30*^a^	88	0.22^ab^
3	171	0.44*^bc^	36	0.09*^a^	116	0.30*^a^	68	0.17^ab^
4	192	0.49*^bcd^	34	0.09*^a^	112	0.29*^a^	54	0.14*^b^
5	209	0.53*^cd^	32	0.08*^a^	107	0.27^a^	43	0.11*^b^

*Note*. All post hoc-analyses were Bonferroni corrected (*α* = 0.001). Asterisks based on the estimations of *z*-values indicate significant differences in prevalence among similar waves *between* the cohorts. Hereby, *z*-values below −3.023 and above 3.023 indicate that the differences are below the *p*-value of .05 in a two-tailed test. Prevalence rates sharing the same superscript(s) among the waves are not significantly different from each other *within* the cohorts. This was tested using a confidence level of 99.75% in which non-overlapping confidence intervals indicate significant differences in prevalence rates among the waves.

Our results indicated that a harmonious relationship was the most common throughout adolescence (rates between 30 and 53%). In addition, both uninvolved-discordant and turbulent relationships were relatively common throughout adolescence (rates between 16 and 30% and between 11 and 29%, respectively). The authoritative relationship was also relatively common during early-to-middle adolescence (rates between 21 and 36%), but less common during middle-to-late adolescence (rates between 8 and 11%).

The overall prevalence of each profile differed significantly between the cohorts. Specifically, there were significantly higher rates of turbulent and authoritative relationships and significantly lower rates of harmonious and uninvolved-discordant relationships in early adolescence than in late adolescence. We also identified within-cohort differences in prevalence among the waves. During the early-to-middle adolescent cohort, the prevalence of turbulent relationships significantly increased and the prevalence of authoritative relationships significantly decreased. Furthermore, in the middle-to-late adolescent cohort the prevalence of harmonious relationships significantly increased whereas the prevalence of turbulent relationships significantly decreased. However, no significant changes emerged in the prevalence of a harmonious relationship during early-to-middle adolescent cohort and in the prevalence of the uninvolved-discordant profile throughout adolescence.

### Individual Differences in Development

In the present study, we particularly focused on the transitions across 4-years as they illustrate long-term relationship developments. Table 4 display the transition probabilities of parent-adolescent relationship profiles for early-to-middle and middle-to-late adolescents across a 4-year interval. However, we also provide transition probabilities across a 1-year interval (i.e., short-term developments) in Table 1 of the supplemental material. An important difference is that there was less relationship stability across a 4-year interval when compared to the 1-year interval. Other than that, the most common transition patterns across 1-year and 4-year were relatively similar to each other. In addition, we examined differences in transitions within the profiles and between the cohorts. In the next sections, we describe the transition patterns of adolescents’ perceived relationship quality with their parents that can explain the change and stability in aforementioned relationship quality prevalence patterns.

**Table 4:** Transition probabilities of parent–adolescent relationship change across 4-year interval for young and old cohort

Relationship type in year N	Transition probabilities for parent-adolescent relationship type in year N+4			
	H	A	U	T
Early-to-middle adolescents (N = 919)				
Harmonious (H)	0.52*^a^	0.15*^b^	0.19^b^	0.13*^b^
Authoritative (A)	0.31^a^	0.35^a^	0.11^b^	0.23*^ab^
Uninvolved-discordant (U)	0.27*^a^	0.06*^b^	0.45^ac^	0.22*^a^
Turbulent (T)	0.10*^a^	0.14*^a^	0.13*^a^	0.63*^b^
Middle-to-late adolescents (N = 392)				
Harmonious (H)	0.78*^a^	0.05*^b^	0.15^bc^	0.02*^b^
Authoritative (A)	0.53^a^	0.38^ab^	0.08^bc^	0.02*^c^
Uninvolved-discordant (U)	0.55*^a^	0.02*^b^	0.39^a^	0.03*^b^
Turbulent (T)	0.26*^a^	0.06^*b^	0.36*^a^	0.32*^a^

*Note*. All post hoc-analyses were Bonferroni corrected (*α* = 0.002). Asterisks based on the estimations of *z*-values indicate significant differences in transition probabilities among the profiles *between* the cohorts. Hereby, *z*-values below −2.955 and above 2.955 indicate that the differences are below the *p-*value of .05 in a two-tailed test. In addition, transitions sharing the same superscript(s) in rows are not significantly different from each other *within* the cohorts. This was tested using a confidence level of 99.58% in which non-overlapping confidence intervals indicate significant differences in transition probabilities among the profiles

#### Early-to-middle adolescence

We revealed transition patterns that may explain the decrease in authoritative relationships during early-to-middle adolescence and the low prevalence of this relationship in middle-to-late adolescence. Additionally, we identified transition patterns that explain the increase in turbulent relationships during early-to-middle adolescence.

- Adolescents in an authoritative relationship were unlikely to remain in this relationship as such (only 35% did). Most of those in authoritative relationships changed into a different relationship profile. Specifically, 31% changed into a harmonious relationship, 23% changed into a turbulent relationship, and 11% changed into an uninvolved-discordant relationship profile. Individuals in other relationships were unlikely to change to an authoritative relationship (rates between 6–15%). However, they were still significantly more likely to do so in early-to-middle adolescent cohort when compared to middle-to-late adolescent cohort (rates between 2–6%).
- Adolescents in a turbulent relationship showed high probabilities to remain in this relationship (i.e., 63%). In addition, 13–23% of adolescents in other relationship profiles were likely to change into a turbulent relationship profile. At the same time, those in a turbulent relationship profile were unlikely to change into other relationship profiles (10–14%).

#### Middle-to-late adolescence

We identified transitions that seem to underlie the significant decrease in turbulent relationships and the significant increase of harmonious relationships in middle-to-late adolescence.

- Those in a turbulent relationship showed low levels of relationship stability (i.e., 32%). Of those who changed, 36% of adolescents in a turbulent relationship profile changed into an uninvolved-discordant and 26% of these adolescents shifted to a harmonious relationship. Additionally, adolescents in one of the other relationships were very unlikely to shift into a turbulent relationship (rates between 2–3%). These stability and transition estimates were significantly lower during the middle-to-late adolescent cohort than during the early-to-middle adolescent cohort.
- Adolescents in a harmonious relationship were likely to remain in this relationship (i.e., 78%). Of those who changed, 15% changed into an uninvolved-discordant relationship and only 2–5% of these adolescents shifted into an authoritative or turbulent relationship. Moreover, adolescents in other relationship profiles were likely to shift to the harmonious relationship profile (rates between 26–55%). The high stability of and transitions into a harmonious relationship were significantly higher in the middle-to-late adolescent cohort than in the early-to-middle adolescent cohort.

#### Transitions explaining the non-significant prevalence changes

Finally, we describe transition patterns that may explain the non-significant changes in the prevalence of harmonious relationships during early-to-middle adolescence and the prevalence of uninvolved-discordant relationships during middle-to-late adolescence.

- Although adolescents in a harmonious relationship profile were likely to change into one of the other relationship profiles during early-to-middle adolescence (rates between 13–19%), those in an authoritative (i.e., 31%) or uninvolved-discordant (i.e., 27%) relationship were also very likely to change into a harmonious relationship profile.
- During early-to-middle adolescence, 27% of those classified in an uninvolved-discordant relationship profile changed into a harmonious relationship profile and 22% changed into a turbulent relationship profile. However, adolescents of the other three profiles were also likely to shift into an uninvolved-discordant relationship (rates between 11–19%).
- During middle-to-late adolescence, those in the other relationship profiles remained likely to shift into an uninvolved-discordant relationship (rates between 15–36%), whereas 55% of the adolescents in an uninvolved-discordant relationship mainly changed into a harmonious relationship.

In short, the balance between relationship profile shifts in and out of profiles seemed to explain the non-significant change in the prevalence rates.

## Discussion

This study provides the first longitudinal person-centered investigation of the extent to which parent-adolescent relationship quality development is consistent with the separation-individuation, evolutionary, maturational, and realignment perspectives. Although prior person-centered research revealed meaningful individual difference in patterns of relationship development, these studies (Choe et al. 2014; Seiffge-Krenke et al. 2010) lack information using all the key components support, negative interaction, and power, and the extent to which adolescents remain or change from a particular relationship quality profile into another across years. Our study addresses these limitations by applying a LTA procedure using a two-cohort large-scale longitudinal dataset (N = 1311) with five annual waves to examine how adolescents’ perceived relationship quality with their parents changed across years. Findings suggest that from ages 12 to 16 years only a subgroup of adolescents moved away from perceiving an authoritative relationship with their parents or changed into an uninvolved-discordant or turbulent relationship. Interestingly, some continued to perceive an authoritative relationship and many changed into perceiving a harmonious relationship with their parents. From ages 16 to 20 years, a majority of adolescents changed the relationship with their parents into a harmonious one. However, some continued to perceive the relationship with their parents as uninvolved-discordant or turbulent.

Together, our results seem to partly provide support for the maturational and realignment perspectives in terms of adolescents’ perceived relationship development with their parents. Specifically, partly in line with these perspectives, we found evidence that only some adolescents temporarily perceive distress in the relationship with their parents as their relationship evolves from hierarchical into egalitarian. Moreover, we found substantial individual differences indicating that some adolescents do not experience relationship quality development in a way that would be proposed by theoretical notions. Our promising findings shed light on the importance of studying individual differences in relationship development across adolescence. We discuss these findings below.

### Parent-Adolescent Relationship Profiles

Using the key components power, support, and negative interaction, we identified *harmonious, authoritative, uninvolved-discordant,* and *turbulent* parent–adolescent relationship profiles^1^ that only partly overlapped with a prior relationship typology (Hadiwijaya et al. 2015). Similar to this typology, we obtained and replicated the harmonious and turbulent relationship profiles. Unlike the prior typology, we did not obtain or replicate the average relationship profile, but identified two additional profiles (i.e., authoritative and uninvolved-discordant). Specifically, the previously uncovered average relationship seems to be divided into an authoritative and uninvolved-discordant relationship profile. This may be due to slightly different patterns of heterogeneity in our sample related to including a more extensive age range (ages 12–20 year-olds) when compared to the sample that was assessed in previous research (12-years *and* 16-year-olds). The specific profiles that we identified thus seem to be slightly different depending on the sample we examined. Nevertheless, we found substantial replication of these profiles and we argue that the use of profiles is important as it represents *one way* to identify individual differences in relationships while taking account of the *multidimensional nature* of relationships (i.e., constellations of key relational dimensions).

### Development of Parent–Adolescent Relationships Across Adolescence

From ages 12 to 16 years, two important *global* changes emerged. First, there was a steep decline in adolescents’ perceiving authoritative relationships. Specifically, a subgroup of adolescents who perceive an authoritative relationship with their parents were very likely to change to one of the other relationship profiles. This indicates that substantial numbers of the early adolescents moved away from relationships in which perceived support from parents was coupled with perceived parental authority. These findings are also consistent with literature demonstrating that the sharpest decrease in the endorsement of parental authority occurs during early adolescence (e.g., Darling et al. 2008). Second, the prevalence of adolescents’ perceiving turbulent relationships increased. Adolescents in turbulent relationships with their parents typically remained to perceive this relationship, whereas those in one of the other relationship qualities were likely to change to this relationship type. This suggests that a subgroup of the early adolescents moved toward perceiving a poorer relationship, as they seemed to question the authority enforced by their parents.

Overall, these findings are partly consistent with studies showing that parent–adolescent relationship quality worsened in early adolescence (e.g., De Goede et al. 2009; Keijsers et al. 2011; Tsai et al. 2013). The fact that some adolescents move away from perceiving authoritative relationships and that some change into turbulent relationships thus lends partial support to the separation-individuation, evolutionary, maturational, and realignment perspectives, as these theories all propose that early adolescence is a period in which adolescents generally strive for more independence and distress increases in the relationship with their parents.

Additionally, we detected individual differences in relationship quality development that deviate from the aforementioned global patterns of development and theoretical notions. First, *more than one-third* of those perceiving an authoritative relationship continued to perceive the relationship like this. This suggests that a substantial proportion of adolescents does remain to perceive a relationship in which they experience parental support and endorse parental authority. Thus, although most adolescents perceived themselves striving for more independence and grew less likely to legitimate parental authority, some adolescents perceive themselves as accepting their parents authority to set rules in certain areas of their lives (Darling et al. 2008). Individual differences in the *belief of endorsing parental authority* may explain why some adolescents remained in an authoritative relationship, whereas others moved away from it. This, however, is not necessarily alarming as those who endorse parental authority in a supportive relationship seem to be more likely to voluntarily disclose information to their parents (e.g., Darling et al. 2006). Parental disclosure, in turn, seems to be linked to positive outcomes during adolescence (e.g., Keijsers et al. 2009).

Second, our findings show that *many adolescents* experience improvements instead of difficulties in the relationship with their parents. Specifically, about half (52%) of the adolescents who perceived a harmonious relationship at the beginning of the study remained to perceive a harmonious relationship with their parents. Many others who were initially not classified in a harmonious relationship profile even changed into a harmonious relationship profile (rates between 10–31%). These findings seem to be in line with a previous meta-analysis which indicated that parent-adolescent conflicts generally decreases across years (Laursen et al. 1998). Furthermore, our findings relate to the *modified storm-and-stress* perspective (Arnett 1999), which specifies that only a subgroup perceive difficulties during adolescence. They are also in line with studies demonstrating that only some perceive distress in the relationship with their parents (e.g., Choe et al. 2014; Seiffge-Krenke et al. 2010; Skinner and McHale 2016; Timmons and Margolin 2015), perceive mood disruptions (Dekker et al. 2007), and engage in risk behavior (e.g., Marti et al. 2010). Overall, it seems that only some adolescents perceive trouble in the relationship with their parents while many others do not.

From ages 16 to 20 years, we identified three important *global* findings. First, there was an increasing prevalence of adolescents perceiving a harmonious relationship with their parents. Specifically, adolescents in a harmonious relationship typically remained in this relationship and if those in other relationship profiles changed, they most often changed into this relationship. Second, those who perceived turbulent relationships became less common. Adolescents in a turbulent relationship mostly changed into another relationship type, whereas changes into the turbulent profile were uncommon. Third, adolescents perceiving authoritative relationships remained uncommon in late adolescence. Overall, these findings show that an increasing number of adolescents changed into a relationship in which they perceived support and equality with their parents, whereas a decreasing number of adolescents moved into a relationship in which they perceived conflicts and/or endorsed parental authority. This implies that many adolescents’ perceive restorations or improvements in the relationship quality with their parents by the end of adolescence. Our results seem to be consistent with previous work showing that late adolescents were less likely to legitimate parental authority (e.g., Darling et al. 2008) and that parent-adolescent relationship quality improves by late adolescence (e.g., De Goede et al. 2009; van Wel 1994). The change of many, but not all, adolescents into a harmonious relationship thus seem to relate partly to the maturational and the realignment perspectives, which propose that hierarchical and/or perturbed parent-adolescent relationships generally become egalitarian and supportive.

Furthermore, we also identified individual differences in development in late adolescence that deviate from the theoretical perspectives. A striking example of this is that *not all adolescents* changed to perceive a harmonious relationship with their parents. In fact, more than one-third of the adolescents continued to perceive an uninvolved-discordant or in a turbulent relationship. A substantial subgroup of adolescents thus seems to fail in establishing a satisfactory relationship quality with their parents. This is worrisome, also because of the so-called *cross-relationship continuity* phenomenon (Seiffge-Krenke et al. 2010). This phenomenon entails a long-lasting effect in which adolescents in hostile family environments are susceptible for developing poor quality romantic relationships (e.g., Ehrensaft et al. 2003). Practitioners should bear this in mind when working with late adolescents who perceive a hostile relationship with their parents. Additionally, future studies could examine the extent to which this hostility transfers to other relationships. Note, however, that most adolescents did perceive a satisfactory relationship with their parents by the end of adolescence. This suggests that many may come to experience the cross-relationship continuity phenomenon in a positive way.

Importantly, we also identified considerable relationship stability next to the aforementioned changes. Specifically, 35–63% of early adolescents and 32–78% of late adolescents across all relationship profiles remained to perceive their current profile. This implies that *a substantial number* of adolescents experienced no changes in the relationship quality with their parents across the years. These findings seem to be in contrast to the four perspectives that all assume change in parent–adolescent relationship quality in terms of increasing distress and independence. However, they add to previous literature by indicating not only that some abusive or neglective parent-adolescent relationships (i.e., turbulent, uninvolved-discordant) remain unchanged (e.g., Laursen and Collins 2009), but also that some emotionally close relationships could remain stable as well (i.e., harmonious, authoritative).

In sum, with two cohorts that together covered ages 12–20 years, we identified a reverse U-shape pattern of parent-adolescent relationship development in which some adolescents perceived distress in the relationship with their parents to increase and then to decrease as the relationship with their parents changed from hierarchical to egalitarian. These findings are partly in line with the findings of De Goede et al. (2009). However, we also extend their findings by demonstrating individual differences in relationship development while taking the several relationship quality dimensions into account simultaneously. Furthermore, because some of our findings indicate temporary deteriorations in parent–adolescent relationships, they can be linked to the reverse U-shape pattern found in adolescence in terms of delinquency tendencies (e.g., Moffitt 1993) and aggression (e.g., Meeus et al. 2016). In addition, they relate to the U-shape pattern found in adolescence with respect to moral judgment (e.g., Eisenberg et al. 2005) and empathic perspective taking (e.g., Van der Graaff et al. 2014). However, due to the identification of substantial individual differences, it should be kept in mind that only some adolescents experience their social developments to first deteriorate and then restore later again as they become independent.

### Associations with Multifinality and Equality Concepts

Individual transition patterns shed light on the *multifinality* and *equifinality* concepts of developmental pathways (e.g., Cicchetti and Rogosch 2002). Specifically, multifinality entails that any starting point evolves in diverse final states, whereas equifinality suggests that different starting points develop into one final state.

During early to middle adolescence, we mainly found evidence for multifinality. Although the overall prevalence rates indicate that adolescents systematically perceived a turbulent relationship or moved away from perceiving an authoritative relationship in this period, only a subgroup (13–23% of early adolescents) changed to perceive a turbulent relationship or moved away from perceiving an authoritative relationship (65%). In addition, early adolescents were also likely to change into an authoritative or turbulent relationship, next to changing into perceiving a turbulent or harmonious relationship, than late adolescents were. This suggests that early adolescents showed no evident trend toward changing into one specific profile and that they generally changed into one of the four profiles. Early adolescence thus seems to reflect a period in which increased variations in transitions of perceived relationship quality occur.

During middle to late adolescence, we found evidence for both multifinality and equifinality. Multifinality emerged especially for those in a turbulent relationship. These adolescents either succeeded in changing into a harmonious relationship or failed and changed into an uninvolved-discordant relationship. The latter is important as it suggests that those in turbulent relationships may fail in establishing an egalitarian relationship with their parents that is satisfactory. This finding seems to be highly in line with the *autonomy-relatedness* model (Grotevant and Cooper 1986), which states that adolescents’ independence is best achieved in the context of close relationships. Particularly adolescents perceiving a turbulent relationship may therefore perceive difficulties in establishing an independent and satisfactory relationship with their parents because of the disruptions in their relationship. Moreover, equifinality emerged in those perceiving a harmonious, authoritative, or uninvolved-discordant relationship. Adolescents perceiving one of these three relationship qualities were all likely to perceive a harmonious relationship by late adolescence. A harmonious relationship therefore appears to serve as an *endpoint* of relationship formation, indicating that adolescents typically move to perceive an egalitarian and satisfactory relationship by late adolescence (Collins and Luebker 1994; Youniss and Smollar 1985).

### Limitations and Suggestions for Future Studies

One major shortcoming of the present study is the use of a single, self-report measure to examine parent–adolescent relationship quality development. We only provided *perceptions* of adolescents’ relationship developments and lack of information about parental experiences. On the other hand, because relationship quality is mostly in the “eye of the beholder” (e.g., Branje et al. 2002), it is adolescents’ relationship experiences that are crucial in predicting their developmental outcomes (e.g., well-being, self-esteem, academic achievements). Nevertheless, future research should examine whether parents perceive similar patterns of relationship quality development, or investigate how perception similarities and discrepancies in relationship quality evolve throughout adolescence, and affect adolescent and parental adjustment.

Another drawback is that the present study is that *reasons for the observed changes* remained unexamined. For example, it remains unclear why some adolescents perceive a poor relationship during early adolescence, whereas others do not. For example, those who experience more depressive symptoms may be more likely to perceive a poor relationship and would be less likely to change into a satisfactory relationship across years when compared to those experiencing less depressive symptoms (e.g., Branje et al. 2010). Future studies should examine variables that may affect differences in relationship quality development.

Moreover, the present research covered the period of adolescence using a *two-cohort five-wave longitudinal study design* (i.e., 12–16 years and 16–20 years) rather than following the same adolescents from ages 12 to 20. Although early-to-middle adolescents at T5(i.e., average age of 16 years) showed a small difference in their levels of support, negative interaction, and power from middle-to-late adolescents at T1 (i.e., average age of 16 years) both cohorts are quite comparable for two reasons. Firstly, we found the same relationship profiles in both cohorts. Secondly, developmental patterns of mean level change of relationship dimensions were very consistent across both cohorts. Specifically, the decrease in relationship quality reaches its peak in middle-adolescence. That is, the lowest level of relationship quality was found in waves 4 and 5 of the early cohort and in waves 1 and 2 of the late cohort. Similarly, parental power decreased regularly across cohorts, with the smallest differences in power between the fifth wave of the early cohort and the first wave of the late cohort. This consistency across cohorts in mean level change of relationship dimensions is also nicely visible in the prevalence patterns of the relationship types shown in Fig. 2. Thus, we observe systematic developmental trends across both cohorts for each of the four relationship types. Data of mean-level change of the three relationship dimensions can be obtained from the first author.

Finally, examining early-to-middle and middle-to-late adolescence only offers a limited understanding of the timing on relationship quality change and stability patterns that can reach far back into childhood or reach further into adulthood. For instance, those who remained in a harmonious relationship across years may already have had a turbulent phase with their parents in the childhood period. Additionally, those who were in an uninvolved-discordant or in a turbulent relationship by the end of adolescence may just postpone the reestablishment of a satisfactory relationship with their parents into the adulthood (e.g., adolescents who left their parental home) (e.g., Seiffge-Krenke 2013). Future studies should examine relationship quality development covering both childhood and adulthood using one cohort.

## Conclusions

Our study is the first to simultaneously test the separation-individuation, evolutionary, maturational, and realignment perspectives and to demonstrate both typical and atypical individual patterns in adolescents’ perceived relationship quality development by applying a person-centered approach. This is a major contribution since prior studies were mainly variable-centered, included a singular relational aspect, and focused on a general pattern of relationship development only. Although prior person-centered studies revealed meaningful individual relationship trajectories, they lacked information about parent–adolescent relationship quality using all the key components support, negative interaction, and power, and the extent to which adolescents remain or change from a particular relationship status into another across years (Choe et al. 2014; Seiffge-Krenke et al., 2010). Our study has now addressed these shortcomings by applying LTA. We believe that the use of LTA is important as it can provide individual development in detail while taking account of the multidimensional nature of relational concepts. Using this procedure, we demonstrated the need to recognize that although adolescents engage in similar normative developmental tasks (i.e., strive for independence); there are also relationship changes unique to particular parent–adolescent relationship qualities. Our promising findings mark the need for studying individual differences in relationship development across adolescence.

Our study provides new and unique evidence for adolescence being far less intense than presumed, as only a minority of adolescents experienced distress in the relationship with their parents. Importantly, we showed that only some adolescents continued to perceive themselves as dependent upon their parents and that only some ended to perceive a deteriorated relationship. Many adolescents, however, successfully grew to perceive themselves as independent individuals and simultaneously established a satisfactory relationship by the end of adolescence despite the distress that emerged. Thus, only some adolescents experience their independence to bloom after a temporary period of storm-and-stress with their parents.

## Electronic supplementary material

Supplementary Material

## Footnotes

1 Please note that despite the arbitrariness of our profile labels, our profiles do reflect relative tendencies of people classified in these profiles. For example, adolescents in a turbulent relationship are relatively more likely to perceive “turbulence” (i.e., low levels of support and high levels of negative interaction and power) in the relationship with their parents than those in the other relationships.
