# Supplementary material for: On the Development of Harmony, Turbulence, and Independence in Parent–Adolescent Relationships: A Five-Wave Longitudinal Study
Source: J Youth Adolesc. 2017 Jan 2;46(8):1772–88. doi: 10.1007/s10964-016-0627-7 (PMC5491633; doi:10.1007/s10964-016-0627-7)
Supplement: Supplementary file 1 — Supplementary Material [file 10964_2016_627_MOESM1_ESM.docx]

**Fig. 1**

Parent-adolescent relationship profiles for a five-class latent transition solution based on adolescents’ perceived support, negative interaction, and power in the relationship with their mothers and fathers (*N=* 1,311)

Table 1

*Transition Probabilities of Parent-Adolescent Relationship Change Across 1-Year Interval for Young and Old Cohort*

|  | Transition probabilities for parent-adolescent relationship type in year N+1 | | | |
| --- | --- | --- | --- | --- |
| Relationship type in year N | H | A | U | T |
|  | Early-to-middle adolescents (N = 919) | | | |
| Harmonious (H) | 0.81*^a^ | 0.08*^b^ | 0.08^b^ | 0.03^b^ |
| Authoritative (A) | 0.15^a^ | 0.73^b^ | 0.03^c^ | 0.10*^a^ |
| Uninvolved-discordant (U) | 0.12*^a^ | 0.00^b^ | 0.79^c^ | 0.09*^a^ |
| Turbulent (T) | 0.01^a^ | 0.06^a^ | 0.05*^a^ | 0.87*^b^ |
|  | Middle-to-late adolescents (N = 392) | | | |
| Harmonious (H) | 0.91*^a^ | 0.02*^b^ | 0.06^b^ | 0.01^b^ |
| Authoritative (A) | 0.21^a^ | 0.77^b^ | 0.01^c^ | 0.01*^c^ |
| Uninvolved-discordant (U) | 0.23*^a^ | 0.00^b^ | 0.75^c^ | 0.02*^b^ |
| Turbulent (T) | 0.03^a^ | 0.03^a^ | 0.20*^b^ | 0.74*^c^ |

*Note*. All post hoc-analyses were Bonferroni corrected (*α* = 0.002). Asterisks based on the estimations of *z*-values indicate significant differences in transition probabilities among the profiles *between* the cohorts. Hereby, *z*-values below -2.955 and above 2.955 indicate that the differences are below the *p-*value of .05 in a two-tailed test. In addition, transitions sharing the same superscript(s) in rows are not significantly different from each other *within* the cohorts. This was tested using a confidence level of 99.58% in which non-overlapping confidence intervals indicate significant differences in transition probabilities among the profiles.
